# Fluorescent Biosensors Based on Silicon Nanowires

**DOI:** 10.3390/nano11112970

**Published:** 2021-11-05

**Authors:** Antonio Alessio Leonardi, Maria José Lo Faro, Barbara Fazio, Corrado Spinella, Sabrina Conoci, Patrizia Livreri, Alessia Irrera

**Affiliations:** 1 Dipartimento di Fisica e Astronomia “Ettore Majorana”, Università degli Studi di Catania, Via S. Sofia 64, 95123 Catania, Italy; antonio.leonardi@dfa.unict.it (A.A.L.); mariajose.lofaro@dfa.unict.it (M.J.L.F.); 2 Istituto per i Processi Chimico-Fisici, Consiglio Nazionale delle Ricerche (CNR-IPCF), Viale F. Stagno D’Alcontres 37, 98158 Messina, Italy; barbara.fazio@cnr.it; 3 Istituto per la Microelettronica e Microsistemi, Consiglio Nazionale delle Ricerche (CNR-IMM) UoS Catania, Via S. Sofia 64, 95123 Catania, Italy; 4 Lab SENS, Beyond NANO, Dipartimento di Scienze Chimiche, Biologiche, Farmaceutiche, ed Ambientali, Università Degli Studi di Messina, Viale Ferdinando Stagno d’Alcontres, 98166 Messina, Italy; corrado.spinella@cnr.it (C.S.); sabrina.conoci@unime.it (S.C.); 5 Istituto per la Microelettronica e Microsistemi, Consiglio Nazionale delle Ricerche (CNR-IMM) Zona Industriale, VIII Strada 5, 95121 Catania, Italy; 6 Dipartimento di Scienze Chimiche, Biologiche, Farmaceutiche, ed Ambientali, Università Degli Studi di Messina, Viale Ferdinando Stagno d’Alcontres, 98166 Messina, Italy; 7 Dipartimento di ingegneria, Università degli Studi di Palermo, Viale delle Scienze BLDG 9, 90128 Palermo, Italy; patrizia.livreri@unipa.it

**Keywords:** silicon nanowires, fluorescent sensors, biosensors, light-emission

## Abstract

Nanostructures are arising as novel biosensing platforms promising to surpass current performance in terms of sensitivity, selectivity, and affordability of standard approaches. However, for several nanosensors, the material and synthesis used make the industrial transfer of such technologies complex. Silicon nanowires (NWs) are compatible with Si-based flat architecture fabrication and arise as a hopeful solution to couple their interesting physical properties and surface-to-volume ratio to an easy commercial transfer. Among all the transduction methods, fluorescent probes and sensors emerge as some of the most used approaches thanks to their easy data interpretation, measure affordability, and real-time in situ analysis. In fluorescent sensors, Si NWs are employed as substrate and coupled with several fluorophores, NWs can be used as quenchers in stem-loop configuration, and have recently been used for direct fluorescent sensing. In this review, an overview on fluorescent sensors based on Si NWs is presented, analyzing the literature of the field and highlighting the advantages and drawbacks for each strategy.

## 1. Introduction

Biosensors are each day becoming more and more important, facing the challenge of novel devices to enable selective and high-sensitive monitoring of several diseases with the requirement of an easy, non-invasive, and preferably out-of-hospital analysis [1]. During the last decades, several biomarkers involved in physiological processes emerged as crucial instruments to monitor the health state of a patient in relation to different diseases [2,3,4,5,6] with a massive impact on both biomedical research and clinical applications. Biomarkers offer the advantage of detecting the presence and quantity of specific biomolecules with quantitative methods, measuring the hazard and the health state of the patient. Moreover, another benefit compared to other sensing applications is the relatively easy design of selective probes (that are often already available in nature) for biomarker specific detection.

Several biosensing approaches were studied and carried out over these years, involving different transduction mechanisms, such as electrical [7,8], electrochemical [9,10], colorimetric [11,12,13], chromatographic [14,15], and optical [16,17]. Among all the biosensors, optical platforms have widespread application thanks to their robustness, sensitive measurements, absence of electrical noise, easy sample preparation, and non-destructivity of tissues samples or cells [18,19,20].

In this class of platforms, fluorescent probes are some of the most common ones with the application of different light-emitting molecules for a variety of analyses involving metal ions [21,22,23], proteins [24,25,26], RNAs and DNAs [27,28,29], and so forth. Another advantage of light-emitting assays is the possibility to be coupled with microscopy analyses improving the sensitivity and spatial resolution and allowing analysis in vivo or inside cell cultures [30,31,32]. Indeed, the development of fluorescent probes that can be precisely located inside the cells is a highly demanded point to investigate certain biomolecule target activity revealing their biological roles [33]. So far, several organic fluorophores have been reported in the literature [34,35,36]. One of the main advantages of organic fluorophores is their readily cost-effective availability. However, the fluorescence signal is very sensitive to the solution, is unstable, and can be affected by photobleaching that causes information losses. Moreover, molecules agglomeration can also induce a quenching effect, limiting the detection efficiency and reliability of these probes [37].

Over the last few years, there was an exponential growth in interest in the application of fluorescent nanoprobes based on semiconductor and metal nanostructures. This interest can be ascribed to the possibilities offered by nanotechnology to improve nanomaterial physical properties in combination to the high surface-to-volume ratio of nanostructures, permitting them to overcome the standard sensor limits [38,39,40,41,42]. Among all the nanomaterials, thanks to their robust photostability, efficient fluorescence at tunable wavelength, semiconductor light-emitting quantum dots (QDs) are typically known as highly fluorescent nanoprobes and have been largely applied in imaging, as well as in biosensing applications [43,44,45,46,47,48,49]. Nonetheless, QDs often are characterized by a complex synthesis that results incompatible with industrial processes. Moreover, agglomeration processes in solution affect the use of QDs making often necessary the use of capping agents that can interfere with molecule labeling processes [50]. Additionally, QDs purification after their tailored surface modification is a delicate step, possibilly leading to a relevant material loss or chemical interference.

Another interesting strategy is the use of one-dimensional nanomaterial (as nanowires) coupled with highly specific recognition ligands (organic molecules or biomolecules) for biosensors that can be applied for several targets and even inside the cells [51,52,53,54]. Compared to 0D nanomaterials, 1D structures can be functionalized with different probes for multiple sensing [55] and its specific position can be finely controlled adopting strategies that take advantage of microcontrollers and microscopes [56,57]. For these reasons, several works report the interest of 1D nanostructures to realize highly sensitive and selective sensors for chemo- and bio-targets that can be analyzed even inside a single cell, with interesting applications in intracellular biochemical phenomena [55]. Nanomaterials are also used as a substrate to improve the stability of dyes [52,53,58,59,60] and as fluorescent quenchers in a structure called nano-molecular beacons (nanoMBs) [61,62]. Several nanomaterials, such as carbon nanotubes, graphene, and gold nanoparticles, have been utilized as novel quenchers coupled with light-emitting molecules for the realization of nanoMBs that found a use, for example, as highly sensitive and multiplexed sensors for DNA detection [61,63,64,65]. Currently, nanostructured biosensors are still not sufficient to enter the market. Without focusing on the specific reasons for each device, it is possible to find a common explanation in the high cost of some used materials, often associated with difficult compatibility of several synthesis approaches with the current industrial fabrication processes. For these reasons, due to the silicon leading position in the microelectronics industry, Si nano-sensors emerge as a likely compromise between the nanomaterial advantages and the industrial requirements, paving a possible commercial transfer in the near future.

### Silicon Nanowire Sensing Platform

In the field of silicon nanostructures, silicon nanowires (Si NWs) have shown great potentialities for various applications such as electronics [66,67,68], photonics [69,70,71,72], energy harvesting [73,74,75,76], and bioapplications [57,77,78,79,80]. Considering the interest in 1D nanostructures for biosensing, Si NWs arise as good candidates due to their convenience with integrated circuits and microelectronics, but also due to their nontoxicity, excellent electronic/mechanical/optical properties, surface tailorability, and biocompatibility [81,82,83,84,85]. Moreover, it is reported in the literature that Si NW arrays could also increase the adhesion force between substrate and cell reducing cell spreading [86,87,88].

By being physically locating in a single cell, the 1D fluorescence sensor could avoid the leakage of small molecules and drift of nanoparticles for cell imaging [34]. For silicon nanostructures, Si NWs arose as a competitive alternative as the an-choring substrate to construct sensors for single-cell research [56,89]. In some cases, Si NWs also emerged as a convenient substrate to improve the quantum efficiency of the fluorescent species as reported by Mu et al. [55]. In the past, it has been reported that immobilizing the reactive molecules onto an opportune matrix is an effective strategy to enhance the selectivity and stability of the fluorophores [90]. The coupling of fluorescent species on Si NWs has also been reported as convenient to improve the stability of a fluorophore for certain application. For example, fluorescein is an interesting FDA approved fluorophore and reduced-fluoresceamine has been used for NO detection, but low selectivity and only 2 h stability have limited this application. Miao et al. reported on the covalently functionaliza-tion of Si NWs with reduced-fluoresceamine as a stable fluorescent sensor for NO without the issue of the free fluorophore [91].

Thanks to all these interesting properties of Si NWs, these 1D silicon nanostructures have been utilized in several bio-applications, such as measurement of biochemical activity, gene transduction, biomarker detection, nanoprobes for tumor treatment (i.e., through hyperthermal treatments), and intracellular biomolecules delivery [79,92,93,94,95]. Among all these studies, it is also possible to find the application of nanoparticles (NPs) decorated Si NWs that have been intensively studied as sensor SERS, and as nanoMBs [78,96,97]. The application of Si NWs as a field-effect transistor is the most common and most performing type of Si NW sensor. Si NW FET sensors make possible the detection of single-molecule (protein [98], DNA [41,99]) that is not possible by fluorescent sensors. However, for most of the applications, this limit of detection (LOD) is not required and typically a LOD (and working range) around nM and pM is enough [100]. Moreover, Si NW FET sensors need microelectronics advanced equipment with an expensive and time-consuming fabrication. Despite Si NW FET have the best LOD, fluorescent Si NW optical sensors are a promising candidate for first screening analyses, where fast, easy-to-use, and cost-effective platforms can be used as a rapid initial check before laboratory analyses, and can be coupled in most cases to existing microscope equipments in combination with standard fluorescent molecules.

**Figure 1:**
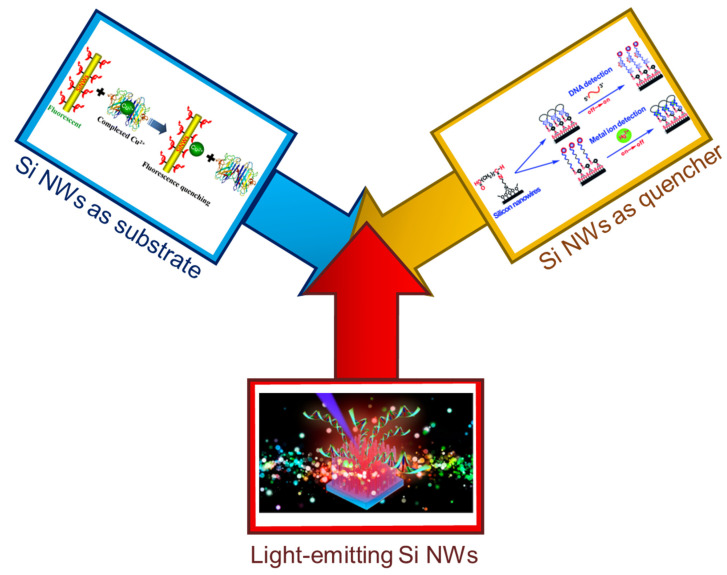
Schematic representation of the main fluorescent sensors based on Si NWs. From the top left corner in clockwise order: Si NWs used as a substrate to improve the photostability of organic fluorophores [60], Si NW quencher in stem-loop DNA configurations [96], and Si NWs as direct light-emitting sensors [102]. The image on the left corner is reproduced with permission [60], Copyright 2014, American Chemical Society. The image on the right corner is reproduced with permission [96], Copyright 2014, Royal Society of Chemistry. The image on the bottom is reproduced with permission [102], Copyright 2018, American Chemical Society.

As far as the fabrication of Si NWs-based fluorescent sensors is concerned, it is possible to generalize 3 types of major Si NW applications as schematized in Figure 1. In particular, (a) Si NWs (or a single Si NW) coupled with fluorescent biomolecules can be employed and used as a substrate [33,34,55,56], (b) metal decorated Si NWs coupled with fluorescent biomolecules can be used as quencher according to the structural conformation of the functionalized material (i.e., quenching when the dye is in close proximitity to gold NP decorated Si NWs) [96,97], (c) light-emitting Si NW directly used as biosensor based on their photoluminescence (PL) quenching [101,102,103]. During the next sections, each one of these interesting strategies will be presented, discussing their major advantages and drawbacks and the type of applications where they have been reported so far.

## 2. Fluorescent Sensors with Si NW as a Substrate

The first reported Si NW fluorescent sensor relies on the use of these nanostructures as a convenient substrate to improve the stability and performance of a fluorescence ligand. In particular, Mu et al. used Si NWs coupled with N-(quinoline-8-yl)-2-(3-triethoxysilyl-propylamino)-acetamide (QlOEt) for the detection of Cu^2+^ [55]. Several metal ions can be found in our body, which are involved in physiological processes. Among them, Cu^2+^ is one of the most abundant with a concentration of 100 μM in healthy cells [104,105] and complexed Cu^2+^ are implicated in important physiological processes as oxidative stress, biocatalysis, and several diseases [106,107,108,109]. Free Cu^2+^ ions are not commonly found inside the body, but chelated by several biomolecules such as tyrosinase, ceruloplasmin, and SOD1 (a copper-binding enzyme, Cu−Zn-superoxide dismutase) [104,105,110]. Hence, the monitoring of the complexed Cu^2+^ in living cells is of great interest for both understanding the Cu^2+^ roles in biological systems and its application as a biomarker [60].

The authors synthesize Si NWs by thermal evaporation and with a 15–25 nm crystalline silicon core surrounded by 1–3 nm of SiO_2_. Si NWs were then functionalized with about 9 × 10^−6^ M QlOEt and it was shown that QlOEt-modified Si NWs had higher fluorescence quantum yield than free QlOEt. The authors through IR measurements report that for QlOEt-modified Si NWs there are unreacted silanol groups present at the surface of SiNWs. An hydrogen bonding can occur between the NH group of QlOet and these uncreacted silanol groups [55,111]. This bonding can partially suppressi the proton transfer from the NH group to the heterocyclic nitrogen atom within 8-acylamidoquinoline framework resulting in the higher fluorescence quantum yield of the Si NW sensor.

QlOEt was chosen due to its chemical structure, sensitive to metal ions. When a metal ion coordinates QlOEt, its fluorescence is modified, and this is the sensing mechanism of the reported Si NWs-based sensor. Cu^2+^ has strong coordination with QlOEt that determines the selectivity of the sensor. QlOEt does not specifically chelate Cu^2+^. However, after titration of various metal ions, the authors observed that the fluorescence change of QlOEt-modified Si NW sensor in presence of Cu^2+^ is at least 4 times higher the one obtained with other metal ions at the same concentration. In particular, at a metal ion concentration of about 10^−5^ M, for Cu^2+^ fluorescence was quenched by about 80% of the starting one. For an equal amount of Zn^2+^, the fluorescence of QlOEt-modified Si NWs increased by 15%, while Hg^2+^, Ni^2+^, Co^2+^, and Fe^2+^ led to a 20% fluorescence quenching. Other metal ions, such as Mg^2+^, Na^+^, Ca^2+^, K^+^, Pb^2+^, and Cd^2+^, produce a negligible fluorescence change of the Si NW sensor. From these results, the authors demonstrated the selectivity of the QlOEt-modified SiNWs for Cu^2+^.

The Si NW sensor shown a limit of detection (LOD) of 10^−8^ M, 2 orders of magnitude better than the one of the optimized Q1OEt without Si NWs. This type of optical sensor design was very interesting and lead to the realization of other several works by the same group.

### Systems for Macro- and Micro-Sensing

**Figure 2:**
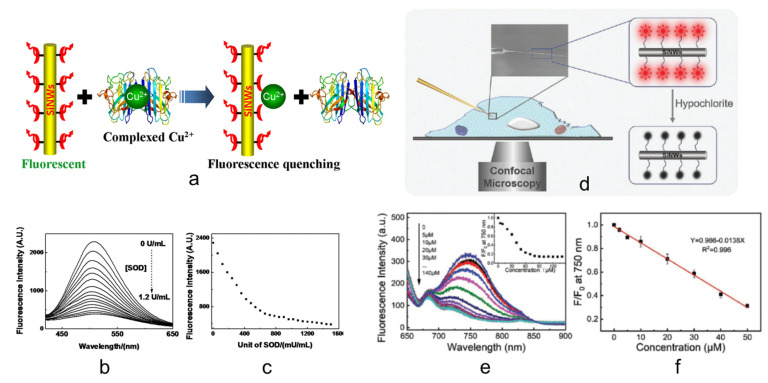
(**a**) Working principle of the Si NW fluorescent sensor for the detection of Cu^2+^ [60]. (**b**) Fluorescence spectra of the Si NW sensor acquired for different concentrations of SOD1 [60]. (**c**) Dose-response curve in terms of fluorescence as a function of mU/mL of SOD1 for the Cu^2+^ detection by the Si NW fluorescent sensor [60]. (**d**) Schematic representation of the HCL fluorescent sensor based on the use of a single Si NW on the tip of a micropipette [34]. (**e**) Fluorescence spectra obtained for different concentrations of HCl. In the inset to e) the complete calibration curve obtained is reported [34]. (**f**) Part of the dose calibration curve where the response can be fitted by a linear trend [34]. (**a**–**c**) are reproduced with permission [60], Copyright 2014, American Chemical Society. (**d**–**f**) are reproduced with permission [34], Copyright 2018, WILEY-VCH.

Miao et al. continue in the Cu^2+^ detection realizing a Si NWs-based fluorescent sensor able to detect this metal ion in its complexed form that can be typically found in the human body. In particular, the sensor was tested with SOD1 and liver extract. In this case, the authors adopted a different functionalization involving a different fluorescent molecule. Miao et al. covalently immobilized on the surface of Si NWs 3-[2-(2-aminoethylamino)- ethylamino] propyl-trimethoxysilane (3-A) as receptor and a dansyl group (D) as a fluorophore. In Figure 2a is shown the final Si sensor (3-AD-SiNW) and its detection mechanism is schematized. In this application, the authors used 2 different approaches for the fabrication of Si NWs. Thermal evaporation was adopted for the realization of Si NWs with about 6–9 nm Si core and 3–5 nm of surrounding SiO_2_ while a metal-assisted chemical etching (MACE) was carried out for the realization of high density and vertically oriented Si NW arrays with bigger Si NWs. The Si NWs were realized by MACE for array applications or for a single Si NW sensor.

The fluorescence signal of 3-AD-SiNWs decreases increasing the Cu^2+^ concentration with a linear dose-response in the 50−400 nM range and a detection limit of about 31 nM. Concerning the selectivity, the authors report on a high binding affinity of the 3-AD-Si NWs for the detection of Cu^2+^ probably due to the suitable radius and electronic structure of the Cu^2+^ for the interaction with 3-A [60]. The binding affinity of the 3-AD-Si NWs was demonstrated by several tests with other metal ions and amino acids that mimes the possible presence of interference molecules. The selectivity has been tested also using a complexing agent as EDTA that produce EDTA-Cu^2+^ complex that owns a high stability constant [112]. The presence of EDTA or its further addition to a Cu^2+^ solution does not change the signal obtained from the Si NW sensor demonstrating that the 3-AD-SiNWs own a high binding affinity with Cu^2+^. The binding affinity of the functionalization was demonstrated by the authors also by using the combination of 3-A and D molecules (3-AD molecules) tested with Cu^2+^ in the supporting information of [60].

Commonly, different amino acids (as Cys, His, Glu, etc) can easily bound Cu^2+^ [105] and the sensor was tested with these and other biomolecules showing negligible interference. All these tests demonstrated the high selectivity of the sensor for Cu^2+^. This Si NW sensor was tested with SOD1, a copper-binding enzyme that is used by our body against oxygen radicals [104], to simulate a real condition where the Cu^2+^ is present in complex form. The sensor perfectly worked in presence of SOD1 with a fluorescence quenching that for each 1.11 U/L SOD1 is comparable to that of 1.0 nM of Cu^2+^. The obtained fluorescence intensity and the calibration curve are shown in Figure 2b,c, respectively. Moreover, the sensor was tested in a more complex (and real) matrix to prove its affordability. In particular, since Cu overload can be found in the liver [104] the authors tested the 3-AD-SiNWs to detect the Cu^2+^ (in its free form and complexed) in prepared mouse liver extract demonstrating a linear response of photoluminescence (PL) quenching to the liver extract. Finally, the authors shown a chip sensor as an array of functionalized Si NWs for the detection in real-time and in situ of free and complex Cu^2+^ inside apoptotic HeLa cells.

H_2_S and NO are two of the main endogenous gaseous transmitters in the human body. H_2_S has a critical role in various physiological processes, in regulating intracellular redox, and in disease signaling processes [113,114]. Wang et al. report on the realization of a Si NW sensor functionalized by naphthalimide azide derivative for H_2_S detection [18]. The authors demonstrated the selectivity of the sensor for H_2_S among other biologically reactive sulfur, oxygen, and nitrogen species. A linear dose-response curve in the 0–40 mM concentration range was found. Moreover, a H_2_S detection in real-time and in situ monitoring from HeLa cells was reported [18]. 

For what concern NO, as endogenous gas the nitric oxide has an important role as a cardiovascular and nervous systems messenger [115,116]. Miao et al. realized a Si NW fluorescent sensor for NO detection based on the reduced-fluoresceamine light emission [91]. In this case, Si NWs are used to improve the selectivity and the stability of the fluorescein which is commonly used as a direct sensor in these applications. The sensor was tested with other endogenous gas and reactive species demonstrating its selectivity for NO detection. In this case, the reaction with the NO causes the turn-on of the light emission of the sensor (and not a quenching). A working range of 0–2.2 mM was obtained and the possibility to operate in a real matrix as liver extract was obtained. Moreover, the authors shown the possibility to obtain a fine spatial resolution by using a single Si NW-based sensor [91].

Another sensor based on the coupling of Si NWs and the fluorescein group was designed by Wang et al. to detect the alkaline phosphatase (ALP) [33]. An anomalous and high concentration of this enzyme in human serum is usually associated with several diseases such as liver dysfunction, breast, and prostatic cancer, etc. [117,118]. A high selectivity among other enzymes and biomolecules along with a linear dose-response curve in the 0.0175–0.3 U/mL range of ALP were obtained. Moreover, the authors shown high spatial resolution analysis by using a single NW sensor for intracellular measurements [33].

Cao et al. report on the fabrication of a Si NW sensor for an important reactive species, hypochlorous acid (HClO) [34]. HClO plays a critical role against pathogens in our body [119,120] and can be also found in dissociate hypochlorite ion form (ClO^-^) [121]. A high concentration of HClO can be associated with different important diseases such as cardiovascular disease and cancer [122,123,124]. The authors fabricate a Si NW sensor for HClO based on the functionalization of nanowires with IR780 as a fluorescent ligand. In particular, the authors show the HClO sensing inside living cells using a micromanipulator to insert a single NW sensor inside HeLa cells, as depicted in Figure 2d. Si NWs were realized by Chemical Vapor Deposition (CVD), for in vitro experiments, and MACE for array experiments, and the single NW sensor application. The sensing mechanism is due to the one-electron oxidation [125], probably resulting in the cleavage of the polymethine chain in IR780 that causes the quenching of the fluorophore light emission as a function of its concentration. The PL quenching obtained increasing the concentration of HClO from 0 to 140 µM is shown in Figure 2e and its inset. A linear response curve was found for the working range of 0–50 µM, as shown in Figure 2f. The selectivity was demonstrated by testing the sensor with some of the main metal ions and reactive species that can be present in the human body reporting a negligible signal variation. Si NW sensors were successfully tested not only with the direct target recognition, but also in a system that mimes the HClO production of our enzymatic system [126,127], demonstrating the ability to detect hypochlorite in living organisms. As schematized in Figure 2d, the authors reported on the possibility to load the tip of a micropipette with a single Si NW sensor that can be micromanipulated to be inserted inside cells for fluorescence imaging through confocal microscopy analysis [34]. This result opens the route to the intracellular application of a single Si NW sensor that can be used in intracellular systems.

Concerning the detection of other important metal ions in our body, Chen et al. realized a Si NW-based fluorescence sensor for Ca^2+^ [56]. Calcium ion is an important messenger in cells, and it is related to several cellular activities and fundamental processes in neurons [128,129]. In this case, the sensor work as a ratiometric sensor based on the use of two fluorescent species, a ruthenium-based dye with red emission as the reference molecule, and the Fluo-3 with green emission as the light-emitting probe. The selectivity was demonstrated with respect to several metal ions and reversible detection of Ca^2+^ is reported. The authors also demonstrate in this case the use of a single Si NW fluorescent sensor loaded in the tip of a micropipette for intracellular sensing. In particular, by a micromanipulator and confocal microscopy analysis, the sensor was able to recognize the difference between the Ca^2+^ concentrations in the cell body and the neurites with a high spatial resolution [56].

The use of Si NWs as a substrate to improve the stability, selectivity, and sensitivity of different fluorescent species, and for different applications has been highlighted. In Table 1 all the sensors discussed in this second section are reported highlihting the probe, targe, matrix, and LOD. The LOD shown by these sensors is commonly in the nM–µM range but in any case, able to surpass the direct fluorophore use and enough for real applications. This class of sensors shows great potentiality in biological studies with the possibility to replace standard fluorophores and showing real-time and in situ detection for intracellular analyses. Indeed, the realization and use of a single Si NW sensor capable to be finely located for high spatial resolution imaging of dynamical biomolecules activity is extremely interesting. These Si NWs-based light-emitting sensors may found a commercial transfer as fluorescent probes thanks to their superior performances, their easy use with the current biological equipment (as the confocal microscopy), and the interest in a Si-based platform.

## 3. Fluorescent Sensors with Si NW as a Quencher

Molecular beacons are probe molecules exhibiting a characteristic stem-loop structure where 5′ and 3′ ends are preserved in proximity [130,131]. When the molecular beacon probe hybridized with the DNA target corresponds a structural conformational change that separates the 5′ and 3′ ends. A commonly transducing strategy for these probes is detecting the fluorescence from a fluorophore at one end of the probe that is suppressed by a quencher in the original stem-loop structure before the target capture [130]. Indeed, at the beginning, due to the stem structure, the quencher is very close to the fluorophore suppressing its light emission. Upon the specific hybridization between the target (RNA or DNA) a conformational change in the MBs happens increasing the distance between the quencher and the fluorophore, and so restoring its light emission [96]. The use of molecular beacons is very interesting due to the high selectivity, multiplexed detection capability, and for the possibility to discriminate single base mismatch in DNA.

As elicited in the introduction the use of nanomaterials as quenchers in nanoMBs founds interesting applications in the literature [61,63,64,65]. Au NPs are one of the most used nanostructures for the realization of nanoMBs with a quenching action of 2 orders of magnitude higher than the one obtained by organic quenchers [65,132]. Among all the possible strategies, Au NPs decorated silicon NWs can be used as high-performance quenchers in nanoMBs permitting to obtain highly sensitive and selective sensors with multiplexed capability [96]. AuNP-decorated silicon nanowires demonstrated a high quenching efficiency (QE > 90%). Moreover, the large surface-to-volume ratio offered by the Si NWs, the know-how of silicon surface chemistry, and the interest of a silicon-based platform make these Si NW sensors promising for stem-loop applications. The sensing mechanism is the typical one of molecular beacons. An on–off switch of the light-emission based on the decorated Si NW (quencher) and the fluorescent molecule (fluorophore) distance follows the disruption or formation of the stem-loop configuration in the presence of targets. The high-surface volume ratio of Si NWs is a critical point for the enhanced sensitivity of these sensors, but it is also used to enable multiplexed of different DNA strands [97].

Su et al. report the first example of silicon-based nanoMBs [96]. Arrays of 4 μm length Si NW with about 170 nm diameter were fabricated by MACE approach and further decorated by AuNPs through a reduction reaction [78,133]. The Au NPs decorated Si NWs were then detached by ultrasonic treatment. An advantage of the use of Si NWs compared to free Au NPs is the stability in presence of higher salt concentrations in aqueous solutions. Indeed, in the tested range (10^−2^–10^−1^ M), Si NW nanoMBs are stable while free Au NPs gradually aggregate, increasing the salt concentrations. Stem-loop structures of Si NW nanoMBs with DNA probe coupled with FAM, Cy5, and ROX were realized obtaining more than 90% quenching of the fluorophore emission for each case. The hybridization occurring between the target DNA and the Si NW nanoMBs causes the conformational change of the stem helix with a spatial displacement between the fluorophore and the decorated Si NW. Instead, the increase of fluorophore-Si NW distance decreases the quenching action of the Au NP decorated Si NWs restoring the light emission. As a result, an increment in the acquired light signal is related to the target concentration and a pM LOD was demonstrated. This detection limit is 3 orders of magnitude better than the one of other nanoMBs and free Au NPs [61,65,134]. Another interesting aspect of these nanoMBs is their advantage to discriminate single-base mismatch. Indeed, base mismatch causes a slight difference in the conformational change upon the hybridization, hence causing a slightly different distance between quencher and fluorophore. The signal variation produced by a single-base mismatched DNA can be of about 20% compared to the fully complementary target. This high sequence specificity is very interesting for applications involving mutated genes [61,132,135] and even with a concentration of 10 nM of target DNA, a single-base mismatch can be detected.

Multiplexed detection is a required strategy for several applications such as for the simultaneous detection of several tumor-suppressor genes in early phase cancer [136,137]. The authors demonstrate the multiplexed detection of three types of tumor-suppressor genes (exon segments of p16, p21, and p53 genes) by using 3 DNA probes labeled with FAM, Cy5, and ROX. The use of fluorophore with excitation and emission well separated among them permitted to distinguish the detection of the different targets [96]. These results were promising for the realization of a novel type of silicon-based nanoMBs with pM detection and multiplex analysis capability.

**Figure 3:**
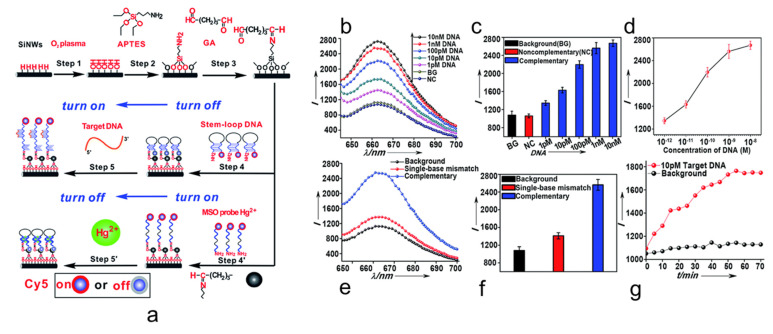
(**a**) Schematic representation of the functionalization steps and working principle of the Si NW nanoMB sensor for DNA and Hg^2+^ detection [97]. (**b**) PL spectra obtained at different DNA target concentrations, background (BG), and non-complementary DNA (NC) [97]. (**c**) PL integrated signal for the different concentrations of DNA target, background, and non-complementary DNA [97]. (**d**) Dose-response curve obtained as the integrated PL signal as a function of the DNA concentration for the complementary DNA. [97] (**e**) PL spectra reporting the comparison between complementary DNA and single-base mismatch DNA signal [97]. (**f**) Integrated PL reporting the clear difference between complementary target and single-base mismatch DNA [97]. (**g**) Kinetic of the fluorescence restoring as a function of the conformational change of the Si NW nanoMB sensors [97]. Figure reproduced with permission [97], Copyright 2014, Royal Society of Chemistry.

Xie et al. demonstrated the realization of Si NW nanoMBs for stem-loop DNA sensing with multiplexing detection for DNA and metal ions as Hg^2+^ [97]. In Figure 3a, the schematic diagram of Si NWs-based nanoMBs functionalization and sensing mechanism is reported for DNA and Hg^2+^ detection. Si NWs were fabricated by MACE and after a functionalization involving glutaraldehyde (GA) and APTES (silane-treatment) are ready to be assembled with the DNA probes tagged with fluorophores [138]. Si NWs guarantee more than 90% of quenching efficiency in the closed stem-loop configuration for DNA and the interaction with the DNA restores the light emission. On the contrary, the sensing mechanism is the opposite for metal ions. For Hg^2+^ detection, after the GA and silane-treatment Si NWs were modified with Cy5-tagged thymine-rich DNA Strands. In this case, the presence of Hg^2+^ ions favors the closing of the DNA structure by thymine–Hg–thymine base pair formation causing the PL quenching of the Si NW nanoMBs [139,140,141]. As shown in Figure 3b the authors demonstrate a wide range of DNA detection from 1 pM to 1 nM. Moreover, in the paper is discussed the possibility of a naked eye detection (just as an on-off signal) with a high concentration of 1 mM for easy-to-use devices for early analysis applications. The sensor demonstrated high selectivity as reported in Figure 3c. The calibration curve obtained for DNA is shown in Figure 3d with an almost linear dose-curve response. The capability of these Si NW nanoMBs to discriminate single-base mismatch was also reported as visible by the PL quenching in Figure 3e and integrated in Figure 3f. In particular, by the integrated PL is possible to observe a variation of about 72% of signal between complementary target and single-base mismatch. The kinetic of light signal restoring due to the capture of the specific DNA target is reported in Figure 3g with saturation in 1 h. Multiplexing capability can be achieved also in this case by modifying the Si NW with different fluorophore tagged DNA probes. The authors demonstrated the multiplexed detection of three tumor suppressor genes by using Cy5, ROX, and FAM. For what concerns the detection of Hg^2+^ a working range of 5 pM-5nM was reported. The selectivity was tested with different more than other 11 interfering metal ions such as Ca^2+^, Cd^2+,^ Mn^2+^, Ni^2+^, Fe^2+^, etc., with negligible interference in Hg^2+^ detection.

The use of Si NWs as a quencher in a stem-loop DNA nanoMBs platform demonstrated improved stability in salt aqueous solution, high-quenching efficiency, and multiplexed analysis capability with an industrially compatible platform. Table 2 shows all the discussed Si NW nanoMBs results reporting the probe, targe, matrix, and LOD.

The LODs shown by these sensors are commonly in the nM–pM range with an improvement of 3 orders of magnitude compared to the most common Au NPs-based nanoMBs. This class of sensors shows great potentiality in biological studies involving DNA base mismatch permitting to resolve even a single base mismatch compared to the complementary target. These Si NW-based light-emitting sensors can be realized with low-cost approaches [96,142,143,144] coupling improved performances to the interest of a Si-based platform and so resulting very interesting for DNA detection and studies.

## 4. Fluorescent Si NW Sensors

Light-emitting Si nanowire recently emerged as an interesting route in the field of fluorescent Si NWs-based biosensors. Silicon is an indirect bandgap semiconductor but it is possible to obtain light emission from 0D or 1D nanostructures with suitable dimensions for the quantum confinement effect. The realization of silicon quantum dots or nanocrystals is extensively reported in the literature due to their high surface-to-volume ratio along with their bright, stable, and tunable luminescence [145,146]. On the contrary, the fabrication of room temperature (RT) light-emitting Si NWs is scarcely reported due to the complex synthesis to obtain these smaller diameters and a high aspect ratio. In particular, this can possibly be ascribed to the difficulties of standard nanotechnology approaches to fabricate Si NW with core dimensions smaller than 10 nm, as required to obtain quantum confinement effect [142]. Recently the use of a thin metal layer in metal-assisted chemical etching (MACE) demonstrated the realization of quantum confined Si NWs that emit light at room temperature by quantum confinement effect [20,147].

This MACE approach for the fabrication of luminescent Si NWs is compatible with the current microelectronics equipment and cost-effective compared to other methods as deep reactive ion etching coupled with state of art lithography to obtain similar dimensions [142]. In particular, the group of Irrera et al. demonstrate that by thin-film MACE it is possible to obtain quantum confined Si NWs with diameters smaller than 10 nm, and a high density (about 10^12^ NWs/cm^2^). All these aspects drove the first application of these structures as direct fluorescent platforms in the field of Si-based biosensors whose sensing mechanism relies on the NW photoluminescence (PL) quenching due to the non-radiative levels introduced by the captured target.

**Figure 4:**
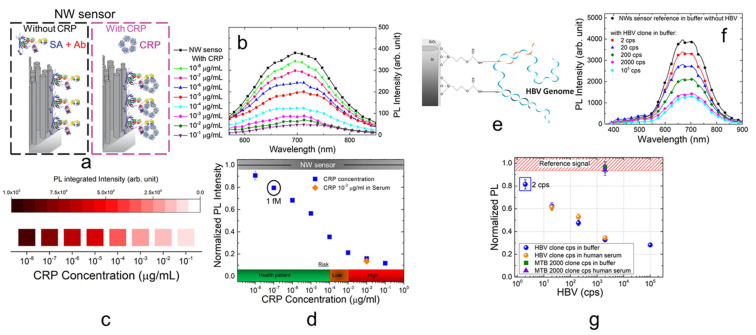
(**a**) Si NW sensor functionalization for the detection of CRP [103]. (**b**) PL spectra as a function of different CRP concentrations. (**c**) Schematic representation of the Si NW quenching (sensing mechanism) as a function of the CRP concentration. (**d**) Dose-response curve obtained as the PL signal of the sensor normalized to the reference signal (obtained without CRP) as a function of the CRP concentration. (**e**) Si NW sensor functionalization for HBV detection [102]. (**f**) PL spectra of the Si NW sensor tested with different copies of HBV in buffer. (**g**) Dose-response curve obtained as the PL signal of the sensor normalized to the reference signal (obtained without HBV) as a function of the HBV copies in 100 µL of buffer (blue points) or human serum (orange points) [102]. (**a**–**d**) are reproduced with permission [98], Copyright 2018, Springer. (**e**–**g**) are reproduced with permission [102], Copyright 2018, American Chemical Society.

In particular, light-emitting Si NW sensors were applied for the detection of different biomarkers such as proteins and DNAs [101,102,103,148]. A Si NWs-based fluorescent sensor was demonstrated for the detection of the C-reactive Protein (CRP), a main cardiovascular biomarker typically measured in blood in the range of 1–100 µg/mL by immunoturbidimetric or other clinical standard procedures [149,150]. The detection of CRP can permit avoiding a myocardial infarction monitoring the health state of a patient. Several studies report on the possible analysis of saliva for a non-invasive analysis that can reduce hospital queue and recovery time pushing the monitoring even at home. However, a working range for CRP concentration of 10^−5^–10^−2^ µg/mL is required limiting the application of standard approaches [151]. In Figure 4a is reported the sensor functionalization and involves the use of the biotinylated specific antibody for the CRP (anti-CRP) linked by streptavidin onto the Si NW surface for the CRP detection. The Si NW sensor was then tested with different concentrations of CRP ranging from 10^−9^ to 100 µg/mL [101]. In Figure 4b, the PL spectra acquired at RT of the sensor tested in the buffer solution without CRP is reported in black. In the same figure, the PL response of the sensor to CRP concentration going from 10^−8^ to 10^−1^ µg/mL is reported. It is possible to observe as increasing PL quenching of the platform for higher CRP concentrations. In these works [101,103], the authors attested through lifetime measurements that the PL quenching can be ascribed to the introduction of new non-radiative levels by the CRP capture, thus reducing the fluorescence. This Si NW PL quenching is the sensing mechanism, and the integrated light signal can be correlated to the CRP concentration as schematized Figure 4c. The calibration curve is reported in Figure 4d showing the integrated PL Intensity normalized with respect to the signal of the sensor without any protein (grey bar) as a function of the CRP concentration. In particular, a detection limit of about 1.6 fM was obtained with a wide operating range. High selectivity was attested in human serum (orange point) and by other negative tests involving other non-specific proteins [103]. This working range permits the application of this label-free sensor for saliva concentration of CRP with a great interest for a point of care non-invasive application. Indeed, the correlated health risk is reported at the bottom of Figure 4d. After the realization of a CRP saliva sensor, by slightly changing the functionalization the same authors demonstrated another platform for blood CRP concentrations [101].

This fluorescent Si NW sensor was also tested for DNA detection realizing a sensor for Hepatitis B Virus (HBV), the major cause of liver disease and liver cancer in the world [152,153]. Quantitative real-time polymerase chain reaction (PCR) is the gold standard for DNA analysis involving the amplification of its number of copies but requires expert personnel, a specialized laboratory and it is expensive in terms of cost and time. Novel detection methods are demanded for applications that does not need the performances of PCR but instead rapid analysis, easier sample treatment, and lower cost [154]. This fluorescent sensor does not require any PCR amplification (PCR-free) or chemical tagging of the target (label-free). In this case, after a silane-treatment of the surface with GOPS the Si NWs were functionalized by using two probes (P1, P2) that are the specific genome sequence complementary to the HBV. The use of two probes complementary for the head and the tail of the HBV genome guarantee a cooperative hybridization to the target improving the selectivity and affordability of the measure. The final sensor and its interaction with HBV is schematized in Figure 4e. In Figure 4f, the Si NW PL response in the buffer to a different amount of HBV cps are reported. The sensor was tested in the typical PCR calibration range from 2 cps to 10^5^ cps over a 100 µL of solution. As for the previous case, increasing the HBV number of copies (cps) follows an increase of the PL quenching. The sensor response was also tested in human serum where a high number of possible interference biological species are present. In Figure 4g, the sensor dose–response curve obtained for both buffer (blue dots) and human serum (orange dots) is shown. This calibration curve was obtained as the integrated PL intensity normalized to the signal acquired by the sensor in the respective matrix (buffer or serum) as a function of the HBV cps. The perfect agreement between the values obtained in buffer and the one obtained in serum is a strong demonstration of selectivity. However, the selective response of the sensor was also demonstrated with a high concentration (2000 cps) of mycobacterium tuberculosis (MTB) as a non-specific target that does not produce any variation compared to the reference signal (sensor without HBV). The sensor shows a remarkable LOD was of 2 HBV cps on the same order as the best quantitative real-time PCR equipment. Finally, the authors demonstrated the use of the sensor with real HBV genome extracted from infected human blood in a buffer matrix. Real DNA has different bases lengths compared to the cloned genome due to the clone realization process. Indeed, the real DNA is about 2 times shorter than the laboratory cloned sequence, and so it may be more complex to detect independently from the transduction method [100]. For real HBV, a LOD of 20 cps was obtained, comparable to the real-time PCR limit.

In Table 3, all the light-emitting Si NW sensors results discussed in this section are reported underlining the probe, targe, matrix, and LOD. This fluorescent Si NW sensing platform shows remarkable performances with a LOD of fM for proteins and few copies for DNA surpassing other fluorescent Si NW strategies. However, it is not the LOD the advantage of this class of sensor. Indeed, compared with other Si NW sensors as Si NWs FET or others state of the art optical platform as photonic crystal, their detection performances are slightly inferior. Despite that, the simple realization, easy-to-use, and cost-effective fabrication of these fluorescent sensors coupled with their performances make them interesting as point of care devices for first analyses outside specialized facilities. The advantage on the other reported fluorescent Si NW sensor is the direct use of light-emitting NWs that does not need to be coupled with other fluorescent species. However, light emission from a single NW has not yet been proven to be enough for single NW fluorescent cells.

In general, the novelty of this platform requires additional tests to attest the sensor reliability. While the other fluorescent Si NW sensor seems to find their best used coupled with the current analysis technologies (as confocal microscopy), this type of fluorescent NW sensor seems promising for the realization of sensing devices that can be widely used outside biomedical laboratories with high sensitivity and selectivity for several biomarker detections. It is important to highlight that as for the other presented platforms, all the reported results were demonstrated in the laboratory. At the moment, a stand-alone portable device able to manage both the Si NW sensor excitation and detection was not reported.

## 5. Future Perspective

All the presented Si NW fluorescent sensors are characterized by advantages and drawbacks. In Table 4, all the main results of this review are summarized reporting the principal characteristics of the discussed sensors such as target, expertise required for the readout, average LOD, and some other interesting information as notes.

Additionally, as future perspective on these technologies, we would like to stress a few keypoints regarding the importance of developing a Si-based sensor which could be easily implemented in the industrial production lines at a low-cost and with outstanding performances arising from the peculiar properties of 1D nanostructures. Indeed, Si NWs offer a huge surface to volume ratio combined with dense array fabrication and a variety of device configurations. The role of Si NW in fluorescent sensors is of great interest and shows huge potential for easy-to-use sensing devices. Nonetheless, possibly due to technological limits, the research in this field is still at the beginning compared to other trasnsductions involving Si NWs (i.e., electrical, electrochemical, etc.). In terms of technological transfer most of the presented state of the art reach the 4th technological readiness level (TRL) achieving the realization of component or board validation in a laboratory. In the future, demonstrating the importance of these technologies will be critical to reach an industrial large scale production for the nanostructures and the realization of a whole system prototype validated in real operational environment.

## 6. Conclusions

In this paper, we have reported an overview on fluorescent sensors based on Si NWs. Si NWs offer the interesting possibility to couple the nanostructure advantages with a Si-based solution. Indeed, the strategic role of silicon in the microelectronics industry combined with the high surface-to-volume ratio and the innovative properties of nanostructures is a promising strategy in the biosensing field.

The fluorescent transduction of Si NW sensor has been explored due to the interest in this sensing mechanism and to the possibility of coupling Si NW fluorescent sensor to the already used fluorescent equipment. In particular, we have presented the most used Si NW-based fluorescent approaches such as Si NWs employed as substrate and coupled with several fluorophores, Si NW quenchers in stem-loop configuration, and Si NW as direct light-emitting sensors. For each platform, the detection performances, advantages, and drawbacks have been discussed. The biosensing field poses exciting challenges still far from being addressed and, in this scenario, fluorescent Si NWs arise as a promising silicon-based strategy.

## Figures and Tables

**Table 1:** Si NW as a substrate coupled with fluorophores.

Probe	Target	Matrix	LOD	Refs.
*N*-(quinoline-8-yl)-2-(3-triethoxysilyl-propylamino)-acetamide (QlOEt)	Cu^2+^	Buffer	10 nM	[55]
3-[2-(2-aminoethylamino)-ethylamino] propyl-trimethoxysilane (3-A) as receptor and a dansyl group (D) as a fluorophore	Cu^2+^	Buffer, liver extract, cell culture	30 nM	[60]
Naphthalimide azide derivative	H_2_S	Buffer, cell culture	mM	[18]
reduced-fluoresceamine	NO	Buffer, liver extract, cell culture	mM	[91]
reduced-fluoresceamine	alkaline phosphatase (ALP)	Buffer, cell culture	0.0175 U/mL	[33]
IR780	HClO	Buffer, cell culture	µM	[34]
Ratiometric detection with ruthenium-based dye as the reference molecule and Fluo-3 as the probe	Ca^2+^	Buffer, cell culture	-	[56]

**Table 2:** Si NW as quencher in nanoMBs.

Probe	Target	Matrix	LOD	Ref.
DNA probe–FAM	Complementary DNA–tumor suppressor genes (p16, p21, p53)	Buffer	50 pM	[96]
DNA probe–CY5	Complementary DNA–tumor suppressor genes (p16, p21, p53)	Buffer	50 pM	[96]
DNA probe–ROX	Complementary DNA–tumor suppressor genes (p16, p21, p53)	Buffer	50 pM	[96]
DNA probe–FAM	Complementary DNA–tumor suppressor genes (p16, p21, p53)	Buffer	10 pM	[97]
DNA probe–ROX	Complementary DNA–tumor suppressor genes (p16, p21, p53)	Buffer	10 pM	[97]
DNA probe–CY5	Complementary DNA–tumor suppressor genes (p16, p21, p53)	Buffer	10 pM	[97]
T-rich mercury-specific oligonucleotide (MSO) tagged with Cy5	Mg^2+^	Buffer	5 pM	[97]

**Table 3:** Light-emitting Si NWs.

Probe	Target	Matrix	LOD	Refs.
Antibody	C-reactive protein	Buffer/Serum	fM	[101]
Primer	Hepatitis B Virus (DNA)	Buffer/Serum	2–20 copies	[102]
Antibody	Small extracellular vesicles	Buffer	10^5^ Ex/mL	[148]

**Table 4:** Comparison between different fluorescent Si NW sensors.

Sensor	Target	Expertise Required	Average LOD	Note	Ref.
Si NWs as a substrate	Metal ions, endogenous gases	Low in macro/High in micro	µM-nM	Can be used for cell imaging	Table 1
Si NW nanoMBs	DNAs	High	nM-pM	Distinguish a single base mismatch	Table 2
Light-emitting Si NWs	Proteins, DNAs, vesicles	Medium	fM for protein, few DNA cps	Does not need other fluoroscent molecules	Table 3

## Data Availability

Not applicable.
